# Finding the answer in space: the mental whiteboard hypothesis on serial order in working memory

**DOI:** 10.3389/fnhum.2014.00932

**Published:** 2014-11-25

**Authors:** Elger Abrahamse, Jean-Philippe van Dijck, Steve Majerus, Wim Fias

**Affiliations:** ^1^Department of Experimental Psychology, Faculty of Psychology and Educational Sciences, Ghent UniversityGhent, Belgium; ^2^Department of Psychology, Cognition and Behavior, University of LiègeGhent, Belgium; ^3^Fund for Scientific Research FNRSBelgium

**Keywords:** serial order, working memory, space, positional models, position marker, spatial attention, hypothesis

## Abstract

Various prominent models on serial order coding in working memory (WM) build on the notion that serial order is achieved by binding the various items to-be-maintained to fixed position markers. Despite being relatively successful in accounting for empirical observations and some recent neuro-imaging support, these models were largely formulated on theoretical grounds and few specifications have been provided with respect to the cognitive and/or neural nature of these position markers. Here we outline a hypothesis on a novel candidate mechanism to substantiate the notion of serial position markers. Specifically, we propose that serial order WM is grounded in the spatial attention system: (I) The position markers that provide multi-item WM with a serial context should be understood as coordinates within an internal, spatially defined system; (II) internal spatial attention is involved in searching through the resulting serial order representation; and (III) retrieval corresponds to selection by spatial attention. We sketch the available empirical support and discuss how the hypothesis may provide a parsimonious framework from which to understand a broad range of observations across behavioral, neural and neuropsychological domains. Finally, we pinpoint what we believe are major questions for future research inspired by the hypothesis.

## Serial order working memory and spatial processing

Working memory (WM) is a fundamental cognitive function and refers to the brief maintenance of information in an active and accessible state such that operations can be performed on it. It is considered to be crucial for major cognitive skills like language, reasoning and learning, not in the least for its core feature of maintaining serial order across multiple items (e.g., Baddeley, 2012). Without the ability to maintain serial order across items in WM, for example, it would be a tremendously effortful job to calculate the overall price of your purchases in a shop, to dial the phone number of a friend, to make yourself a decent sandwich or to construct a line of reasoning. In this paper we present a novel hypothesis on the nature of serial order WM.

The study on how serial order is coded within WM has a strong empirical tradition (Ebbinghaus, 1885 [1964]; Lashley, 1951; Sternberg, 1967; for a review see Marshuetz, 2005) and several theoretical models have been described. Broadly speaking, these models can be divided into two classes: associative chaining and position marker models. Associative chaining was one of the earliest approaches (e.g., Ebbinghaus, 1885 [1964]), the basic underlying idea being that serial order derives from associations between successive items and that each item acts as a retrieval cue for the next item (e.g., Lashley, 1951). Chaining models have been very efficient in explaining a hallmark observation in serial order recall performance: the gradual increase in RT when retrieving order information later in the memorized sequence (e.g., Sternberg, 1967). However, several objections to chaining models can be identified as well. Chaining models have difficulties in explaining the error patterns that are typically observed in serial order retrieval. For example, because it is assumed that order is encoded by contiguous associations between items, recall should fail for items following an erroneous recall. This is not typically observed. In addition, chaining models also have difficulties to explain distance-effects observed in WM (i.e., the observation that it is more difficult to determine the serial order for serially nearby compared to more distant items; Marshuetz et al., 2000; Attout et al., 2014). For (mainly) these reasons, theorists gradually rejected the hypothesis that chaining plays a crucial role in serial order memory (e.g., Henson et al., 1996; Farrell and Lewandowsky, 2002; Burgess and Hitch, 2006) and shifted towards the currently dominant position marker models.

Position marker models build on the idea that serial order coding in WM is achieved by binding the various items to-be-maintained to specific position markers (e.g., begin vs. end items, Henson, 1998; encoding strength, Page and Norris, 1998; oscillatory response, Brown et al., 2000; rank codes, Botvinick and Watanabe, 2007) and that serial order retrieval is achieved by recalling this conjunction. Despite being relatively successful in accounting for empirical observations and despite recent neuro-imaging support for the existence of position markers (Kalm and Norris, 2014), these models are still largely formulated on theoretical grounds and few specifications have been provided with respect to the cognitive and/or neural nature of position markers (but see below on Botvinick and Watanabe, 2007). Here we hypothesize on a novel candidate mechanism to substantiate the notion of serial position markers by relating it to the spatial attention system.

### The mental whiteboard hypothesis on serial order WM

Before we present our hypothesis we start by outlining the empirical background to it, which involves interactions between serial order and spatial processing. First, van Dijck and Fias (2011) asked participants to maintain in WM a series of fruit and vegetable names in the order of presentation, with item presentation always centrally on the screen. It was observed that retrieving early items from this WM sequence facilitated a left hand response, while later items facilitated a right hand response. Importantly, using such *non-spatial* and centrally presented material prevented any confounding with involvement of spatial processing related to stimulus presentation. This study was the first to indicate a close link between serial order in WM and spatial processing.

In a second step, we confirmed that internal selective attention is intrinsically involved in searching through the serial order representation in WM. From the notion that there is direct interfacing between internal and external selective attention (Downing, 2000; Awh and Jonides, 2001; Corbetta and Shulman, 2002; Nobre et al., 2006; Johnson et al., 2013; Kiyonaga and Egner, 2013; Van der Lubbe et al., 2014), we combined a similar WM manipulation as described above with the well-known Posner cuing paradigm—typically used to study (external) spatial selective attention (Posner et al., 1982). In the Posner paradigm, it has been shown that an attention cue (for example a centrally presented arrow) presented shortly before a to-be-detected dot appears left or right on the screen, facilitates performance when it cues the subsequent dot location validly, but impairs performance when it cues the opposite location. We replaced the arrow cues by items (i.e., numbers) that were maintained in serial order WM, and observed that processing of *later* (in time) items of the WM sequence directed attention more to the right than *earlier* items within that sequence (van Dijck et al., 2013). This finding indicates that shifting attention within the internal space for serial order coding can be measured with external attention tools due to their direct interfacing. In a follow-up study, van Dijck et al. (2014) further replicated these findings and extended it to letters as the WM items. The latter was important in order to show the generalization of the mechanism.

Finally, the link between serial order WM and spatial processing was recently shown to also hold in the opposite direction. More specifically, retrieval from serial order WM was found to be facilitated (or hindered) by task-irrelevant, exogenous spatial attention cues (De Belder et al., in revision): exogenously directing attention to the left (right) facilitated retrieval of items early (late) in a WM sequence. Together with the work by van Dijck et al. (2013, 2014) this supports the bidirectionality of these effects, and further strengthens the notion that space is intrinsically involved in serial order WM.

Based on these findings we propose the hypothesis that serial order WM is grounded in the spatial attention system: (I) The position markers that provide multi-item WM with a serial context should be understood as coordinates within an internal, spatially defined system; (II) internal spatial attention is involved in searching through the resulting serial order representation; and (III) retrieval corresponds to selection by spatial attention. To illustrate our hypothesis, one may use a simple analog of a whiteboard: Whenever we need to remember a series of items in a specific order, we represent and maintain these items—for example, from left to right—on a “mental whiteboard”, in strong analogy to writing the items down on a physical whiteboard for later consultation. In fact, this comes very close to what was proposed—but not further specified—by Oberauer (2009, p. 53) who suggested that a “spatial medium of representation [is used] as a projection screen for relations on nonspatial dimensions”—such as serial order. Moreover, like external consultation itself involves moving the putative searchlight of attention (Crick, 1984) across the whiteboard, search and retrieval processes in serial WM are based on (selection by) internal spatial attention. We refer to our hypothesis as the *mental whiteboard hypothesis*.

In principal, the internal space that is used for serial order coding allows for flexibility: Coordinates along any well-arranged and orderly continuum (e.g., left to right; top to bottom; et cetera) may be recruited as best fits the task at hand. For example, in our work reported above, the task involved horizontally arranged stimuli and/or responses, favoring left to right encoding of serial order. With vertical task arrangements, however, top to bottom encoding may be probed (e.g., Dutta and Nairne, 1993; see below). Crucially, when the context does not cue spatial coding otherwise, we assume that it spontaneously occurs from left to right on the basis of the typically observed leftward bias in spatial processing (Jewell and McCourt, 2000; Della Sala et al., 2010) and/or a shaping by reading and writing direction (Whorf, 1956; Maass and Russo, 2003; Spalek and Hammad, 2005; Bonato et al., 2012). Especially reading direction has been shown to be an important force in shaping mental representations of space across related literatures (Zebian, 2005; Shaki and Fischer, 2008; Shaki et al., 2009; Bonato et al., 2012).

### Position marking on the mental whiteboard

#### What does the mental whiteboard hypothesis offer to existing position marker models?

The primary value lies in filling in (some of) the blanks of the precise nature of position markers (i.e., coordinates in internal space) and the corresponding search (i.e., spatial attention) and retrieval processes (i.e., selection by spatial attention). Moreover, as the ground rules of computationally tested position marker models do not change by substantiating markers in terms of spatial coordinates, hallmark serial order observations (Marshuetz, 2005) can still be explained in our mental whiteboard hypothesis—again grounded in spatial attention systems. For example, the *serial position effect* (i.e., gradual increases in response times for items further in the sequence; cf. Sternberg, 1967) can be directly related to the directional consistency of attentional search—typically from left to right—through the spatially defined mental whiteboard representations. Additionally, the *distance effect* (see above) may be related to the observation that in external space processing, discrimination between two stimuli is more difficult when they are positioned at nearby as compared to further locations (e.g., Cave and Zimmerman, 1997; Bahcall and Kowler, 1999). This observation has been assigned to spatial attention interference, and a potential equivalent of this phenomenon in internal space may explain the distance effect in serial order. Indeed, a similar (attentional) interference explanation may hold for so-called *transposition errors* (e.g., Caramazza, 1996); that is, the observation that errors in serial recall often involve switches between serially nearby items. Finally, let us address the observed asymmetry in performance on backward and forward recall (Thomas et al., 2003). Specifically, response time patterns differ between a condition in which a memorized lists of words needs to be reproduced from start to end, and a condition in which the latter occurs from end to start. This type of observation may well be related to attentional search processes. As there is a strong attentional bias towards shifting from left to right (Jewell and McCourt, 2000; Spalek and Hammad, 2005; Della Sala et al., 2010)—which may itself be linked to reading direction (Spalek and Hammad, 2005)—the above mentioned differences in response time patterns may reflect differences in the experience-based development of attentional scanning and its fluency (with more skilled and/or controlled scanning from left to right) and/or continuous tendencies to abort right to left shifting. Future scrutiny of this type of explanations based on the workings of the (internal) spatial attentional system will help to confirm or falsify our mental whiteboard hypothesis.

Interpreting the notion of position markers as coordinates in an internal space may also help to explain why serial-recall sometimes seems to rely on both chaining and positional mechanisms (e.g., Serra and Nairne, 2000; Kahana et al., 2010; Kahana, 2012; Solway et al., 2012). Specifically, spatial coordinates that code for serial order may *over time* (i.e., when the WM representation is sufficiently long maintained) become associated with each other on the basis of spatial contiguity, and these associations may be responsible for chaining-like effects—without positing chaining as the fundamental mechanism underlying serial order.

A final asset of the mental whiteboard hypothesis is that it allows for convergence between general perspectives on WM and serial order. Over the last decades, WM is increasingly conceived as emanating directly from interactions between attention systems and long-term memory (LTM), with attention prioritizing the processing of specific pieces of information available in LTM (Cowan, 1999, 2001; Engle and Kane, 2004; Postle, 2006; Oberauer, 2009; Gazzaley and Nobre, 2012). Hence, in strong analogy to selecting specific information or locations in the outside world (i.e., *external* selective attention), it is assumed that we can attentionally search for, select and maintain in an active state information that is stored in the LTM systems of our brains (Chun et al., 2011; Kiyonaga and Egner, 2013); WM can be said to be equivalent to *internal* selective attention (Kiyonaga and Egner, 2013). In a broader sense, this attention-based perspective relates closely also to resource-based accounts of WM, such as the time-based resource sharing model of WM (Barrouillet and Camos, 2007, 2012; see also Ma et al., 2014). Critically, little effort has been made so far to conceptually embrace serial order in these general, attention-based perspectives on WM. At the same time, most of the serial order models do not make explicit their links to selective attention. The mental whiteboard hypothesis provides a candidate mechanism to close this conceptual gap between general WM perspectives and the specific notion of position markers. This is important because the success of the general, attention-based perspective on WM will—among others—critically depend on efforts to be reconciled with serial order models.

#### What are the alternatives to “finding the answer in space”?

Within the context of our work showing interactions between spatial and serial order processing, there may be two primary, alternative candidate mechanisms to support the notion of position markers. First, Botvinick and Watanabe (2007) proposed to build serial order on existing rank representations in the brain. Specifically, serially presented items can be tagged to fixed rank codes to maintain position—for example, a first item is tagged to the representation of “1” in the brain, a second item to “2”, etcetera (cf. Marshuetz, 2005). These order tags, then, may subsequently drive spatial processing in line with the Spatial Numerical Association of Response Codes or SNARC effect (Dehaene et al., 1993). The SNARC effect involves the robust finding that small numbers are faster responded to with a left hand response, while larger numbers are faster responded to with a right hand response—indicating a link between numbers and space (Dehaene et al., 1993). Currently, this alternative cannot be refuted, but we believe there are some indications in favor of our mental whiteboard hypothesis. In our work on serial order WM we often employed number stimuli, and we systematically observed that number magnitude—often despite its main effect on behavior—did no longer interact itself with spatial processing when casted in a WM sequence (van Dijck et al., 2013, 2014). Additionally, there may be relevant information in exploring serial order and space interactions across the vertical axis. Specifically, whereas number magnitude has been shown to map onto space in a bottom (small) to top (large) direction (e.g., Gevers et al., 2006), Dutta and Nairne (1993) observed a top to bottom organization for serial order WM.

A second alternative mechanism may be based on coding serial order through temporal stamps (e.g., Brown et al., 2000, 2007), with the spatial interactions being a by-effect of the so-called mental timeline (Bonato et al., 2012). Again, such alternative cannot be refuted at the current stance, but tentative indications exist for the claim that space subtends time in serial order coding. Most importantly, van Dijck et al. (unpublished work) show that the impact of serial order on spatial processing reverses when items are serially presented from right to left on the screen. If interactions with space were driven by temporal stamps, then such reversal would not be expected, while it fits well with a flexible system of serial order coding in space. Moreover, van Dijck and Fias (2011) showed that the impact of serial order on space was not due to overall reaction time—and thus elapsed time *per se*—as would be predicted from codes rooted in the temporal domain.

Hence, our mental whiteboard hypothesis builds on the notion that space underlies serial order WM without mediation by either temporal or numerical processes, but future efforts are definitely required to firmly ground this choice.

### Diverse behavioral observations

The studies by van Dijck et al. (2013, 2014; De Belder et al., in revision) demonstrate a clear link between serial order WM and spatial processing along the horizontal axis. Although serial order WM has rarely been linked to spatial processing, the mental whiteboard hypothesis speaks to our imagination: we remember a series of items from left to right on an imaginary bow. This link may help to explain, reinterpret and/or parsimoniously integrate various previous observations.

From the notion that serial order is coded within a spatial coordinate system, a logical next question concerns *how* spatially coded serial order can be combined with other types of spatial encoding. For example, in dance there are tightly integrated temporal and spatial action sequences (i.e., when to perform which movement) that need to be learned and performed—how does the brain achieve this? An answer to this question may be searched in existing literature showing that coding in, maintenance of, and rapid switching between multiple spatial templates is indeed possible (e.g., Derdikman and Moser, 2010; Miles et al., 2011; Nitz, 2012).

At this moment we cannot make any claims about (dis)similarities between spatially coded serial order, and encoding of (external) space *per se*. However, there may be some relevant findings in the literature. From the notion that these build on related spatial systems, a strong prediction would be that interference results from simultaneous encoding and maintaining of serial order and (random) “other” spatial sequences. Hence, if we assume that order information is spontaneously coded along a spatial continuum (e.g., from left to right), then requiring participants to maintain at the same time a sequence of to be remembered information presented on external locations that do not follow this continuum (but rather inhabit space in a random manner), should produce interference because both tax spatial attention in a non-synchronous manner. In fact, various previous studies tentatively suggest that this is indeed the case.

For example, Gmeindl et al. (2011) administered both verbal and visuospatial sequence-memory tasks to participants: They were shown a short sequence of numbers (verbal) or locations (visuospatial) on the screen, and the goal in each trial was to reproduce as many target items as possible either *in the same order* (same order condition) as presentation, or *in any order* (no-order condition). Performance was enhanced in the no-order condition—and this was especially the case for the visuospatial sequences. In a second experiment, Gmeindl et al. again presented verbal or visuospatial sequences to participants. However, now each target sequence was immediately followed by a test sequence of the same kind, and the goal was to decide whether the first and second sequences were identical or not. The second sequence was either identical, contained the same items in a different serial order, or contained a novel item that replaced one of the items of the sequence. It was observed that participants failed to detect changes in serial order between target and test sequences more frequently for visuospatial than for verbal sequences. These findings fit the idea of interference between serial order and (external) spatial coding.

The results by Gmeindl et al. (2011) were more recently corroborated by Delogu et al. (2012). Participants were serially presented with five items (environmental sounds or pictures), and each item was presented at a different location. After this sequence was presented, they were asked to recall a specific item either at the location from which it was presented or at its serial order position within the overall sequence. When participants were instructed to maintain both types of information (i.e., it was not predictable which type of information was required to recall), it was observed for both the auditory and the visual sequences that serial order recall was hindered by the simultaneous encoding of item location, whereas the recall of item location was unaffected by the simultaneous encoding of serial order. This demonstrates again that interference may arise from simultaneous processing of serial order and spatial information, but the asymmetry also provides some information on the possible development of this intrinsic link—with external location information possibly being “prioritized” over serial order coding.

Whereas the findings of Gmeindl et al. (2011) were proposed to indicate domain-specific serial order processes, Delogu et al. (2012) actually interpreted their findings as supporting domain-generality. The latter would indeed be in line with various other studies suggesting domain-generality (Jones et al., 1995; Smyth, 1996; Depoorter and Vandierendonck, 2009), which are discussed in more detail below. For now we would like to point out that the here proposed link between serial order WM and spatial processing could parsimoniously account for these observations together—and this should be further explored in future research.

Another interesting issue is the link between spatially defined serial order representations, and reading and writing. In our culture, the latter develops from left to right and may have contributed to the shaping of spontaneous direction of serial order coding. In fact, *a priori* this was the main reason for predicting spatial coding along the left-right dimension in our previous studies (van Dijck and Fias, 2011; van Dijck et al., 2013). The notion provides some interesting additional hypotheses. For example, one could test a population of participants who read from right to left such as Palestinians or Iranians (Shaki et al., 2009), predicting to find similar but reversed interactions between serial order and spatial processing. Moreover, besides reading from left to right, we also read from top of the page to the bottom. Hence, it may be predicted that serial order WM may also interact with spatial processing along the vertical axis: later items in a WM sequence may trigger attention to be progressively shifted towards the lower regions of a(n) (internal or external) space. Dutta and Nairne (1993) provided some tentative support for this prediction. They presented participants with pairs of items (i.e., shapes), each of which occurred either first or second in time and above or below a fixation point. When both these temporal and spatial dimensions were task-relevant, recall performance was best for sequences in which first and second items were mapped respectively on the top and bottom locations (i.e., congruence between the serial order and spatial domains). In addition to the horizontal axis, this already hints towards a similar link between serial order WM and spatial coding along the vertical axis.

We recently provided more direct support for interactions between serial order processing and vertical processing using a paradigm described by Kirsner and Brown (1981). In their study, Kirsner and Brown (1981) presented participants on each trial with a series of two centrally presented digits which were shortly followed by two digits that were simultaneously flashed, one to the left and one to the right of fixation. Participants had to perform a detection task in which they were required to press a key whenever one of the two lateral digits (the *target*) matched either one of the earlier central digits–thus requiring the maintenance of these earlier digits in WM. Responses were fastest either when a left-side target matched the first presented digit, or when a right-side target matched the second presented digit. Even though this study was not framed as linking serial WM and spatial processing, its results may well be explained as such—providing an alternative to the authors’ original explanation in terms of hemispheric differences. Using this paradigm we show in a recent, unpublished study that serial order processing can interact with spatial processing both across the left-right and top-bottom dimension (Abrahamse, Acar, Fias and van Dijck, in preparation), indicating flexibility in the configuration of spatial coordinates (i.e., position markers) used to maintain serial order. The link to reading is an interesting avenue for future research.

A final issue that we would like to refer to here involves recent support for our hypothesis from research on primates (Adachi, 2014). Elsewhere, we have outlined how our hypothesis on serial order coding in a spatially defined system can account for both the SNARC (van Dijck and Fias, 2011) and attentional SNARC effects (van Dijck et al., 2014). Hence, we postulated that, when performing a typical (attentional) SNARC task, participants may soon form a mental representation in WM that includes the items (e.g., numbers) that occur during the experiment. These items are maintained in WM on the basis of a particular ordinal coding that is probably not the order with which they were presented in during the experiment (as this is not consistent throughout the experiment), but rather follows by default the canonical order implied by number magnitude. As such, serial order effects on response or attention processes may align with magnitude—without magnitude providing the spatial codes itself. Recently, a SNARC-like effect was observed in primates that fits our mental whiteboard hypothesis (Adachi, 2014). Specifically, primates were trained to search for number symbols (i.e., 1–9) within a squared matrix of locations on a screen and touch each of them in a fixed, learned order. Importantly, even though they had no experience with numbers as coding for magnitude (i.e., numbers were meaningless symbols for the primates), it was found that they were faster for items early in the sequence when they were presented left (as compared to right), and vice versa for items later in the sequence. This indicates that, in strong comparison to the human research described above, the left to right spatial coding of serial order information can also be observed in primates (Adachi, 2014, see also Drucker and Brannon, 2014). Interestingly, this finding also informs us that while reading may have contributed to shaping (spontaneous) serial order coding in space in humans, it certainly cannot be the single determinant of these processes.

Overall, even though not unequivocally providing empirical support for our mental whiteboard hypothesis on serial order, the rather heterogeneous set of behavioral studies outlined in this section can be parsimoniously integrated within this single hypothesis.

### Neural substrate of serial order WM

A major challenge with respect to our here proposed mental whiteboard hypothesis concerns (the search for) its neural substrate. While direct investigation is yet to be reported, we would like to discuss two areas that jump out as viable candidates to support this type of coding: the hippocampus and the intraparietal sulcus (IPS).

#### Intraparietal sulcus

The IPS is an area at the lateral surface of parietal cortex. Without claiming its unique and/or sole contribution, specific parts of the IPS have been consistently linked to each of the crucial features that relate to the mental whiteboard hypothesis of serial order WM: verbal (and spatial) STM, serial order processing, and (reorientation of) spatial selective attention. Specifically, whereas more posterior and middle segments of the IPS are systematically involved in selective attention (and the integration of top-down and bottom-up attentional systems), the anterior IPS—especially of the right hemisphere—has been related to serial order coding. This suggests that the IPS provides a neural hub that drives the interactions between serial order and spatial attention. We will elaborate on these issues below and discuss how each of the three crucial features has been related to the others.

Inspired by the multi-component model of Baddeley and Hitch (1974), neuro-imaging studies initially aimed at finding the neural substrates of a dedicated verbal short-term storage system (cf. the phonological loop) that is relatively independent from general attentional processes and other storage systems. This search has not been very successful, as no site seems to respond to these criteria (Buchsbaum and D’Esposito, 2008). Rather, neuro-imaging studies appeared to strengthen the attention-based account of WM. More specifically, the IPS was shown to be sensitive to changes in verbal (Becker et al., 1999; Ravizza et al., 2004; Todd and Marois, 2004; Todd et al., 2005; Majerus et al., 2012) and visuospatial STM load (Nystrom et al., 2000; Majerus et al., 2010), in line with a domain-general (attentional) process that serves both verbal and visuospatial WM (Majerus et al., 2010, 2014; Cowan et al., 2011). Majerus et al. (2014), for example, used a machine-learning algorithm to determine the extent to which common neural patterns characterize WM retention in the verbal and visual modality. They found between-task prediction of the amount of WM load during the retention interval in regions of the dorsal attentional network (posterior parietal and superior frontal cortices), providing novel evidence for common, attention-based neural patterns underlying verbal and visual WM.

The link between IPS and selective spatial attention *per se* has received support though across other studies. Gillebert et al. (2011) reported on two patients that suffered from rare isolated IPS lesions (left posterior IPS vs. right horizontal segment of IPS), and showed its critical contribution to spatial selective attention as these patients were impaired (as compared to controls) on. This neuropsychological evidence confirms earlier indications from neuro-imaging work on the parietal cortex, and especially the IPS (e.g., Corbetta and Shulman, 2002; Hung et al., 2005; Molenberghs et al., 2007, 2008; Vandenberghe and Gillebert, 2009; Silk et al., 2010). Interestingly, Macaluso and Patria (2007) observed IPS activation for attentional reorienting along both the horizontal and vertical axes, while Pavani et al. (2002) showed IPS activation for moving sounds along both horizontal and vertical axes. These observations align with the analog of moving internal attention horizontally and vertically across an internal space.

Activation in IPS has thus been systematically linked both to verbal WM and spatial attention. For our current purposes, this becomes especially interesting in the light of additional neuro-imaging work suggesting that anterior IPS activation subtends serial order coding (Henson et al., 2000; Marshuetz et al., 2000; Majerus et al., 2006). For example, in a functional connectivity study by Majerus et al. (2006), participants performed a verbal WM task that probed recognition for either word identity or word order. They observed consistently stronger right IPS activation for order than for identity information. Left IPS was activated for both types of information, but crucially showed functional connectivity to right anterior IPS only for order information but not for identity information. As such, it could be suggested that (especially right) IPS provides the spatial template to code for serial order information—but possibly the link to verbal items is provided through left IPS. Recent evidence, however, points to the direct involvement of also the left IPS in serial order WM. Using fMRI, Attout et al. (2014) determined the degree of neural overlap between the serial order WM distance effects and numerical tasks and observed *bilateral* IPS activation. Hence, strong hemispheric lateralization of specific functions—if existing in the first place—is yet to be convincingly demonstrated. The link between IPS and serial order is further supported by single-unit recordings in primates (Nieder et al., 2006), as neurons in IPS have been observed to respond selectively to what Botvinick and Watanabe (2007) referred to as rank—which when combined with item information constitutes serial order. Finally, a tentative link to serial order can also be derived from a study by Sakai et al. (2002), who observed right IPS involvement in the learning of finger movement sequences—with motor responses being signaled through spatial stimuli.

Hence, in addition to the already reported roles of IPS in providing an integration zone for multisensory information (Macaluso and Driver, 2005; Anderson et al., 2010), for stimulus-driven and voluntary attentional control (Anderson et al., 2008; Geng and Mangun, 2009), and for temporal orienting and task-specific cortical areas (Davranche et al., 2011), we here propose that IPS is involved in the integration between serial order coding of information in WM and the spatial attention system. Moreover, the systematic links of IPS to both serial order and spatial selective attention supports the hypothesis that serial order maintenance is spatially defined. Future work is needed in order to reveal the full network that underlies serial order WM and internal attention.

#### Hippocampus

The second area that may be proposed as relevant for spatially defined serial order coding is the hippocampus. The hippocampus is traditionally linked to LTM. Specifically, it has been proposed to be involved in the creation and consolidation of episodic memory traces, with a particular focus on spatial information and navigation (e.g., Ekstrom et al., 2003). However, the exclusive link to LTM has recently been reconsidered, with various authors claiming that hippocampus also is involved—one way or another—in short-term and/or WM (Jensen and Lisman, 2005; von Allmen et al., 2013). Most interestingly for current purposes, it has been proposed that hippocampal theta and gamma oscillations together provide a system for serial order coding in WM (Jensen and Lisman, 2005). Specifically, a group of cells representing a single item is proposed to fire on each theta cycle, but only in a given gamma subcycle—thereby providing a short-term buffer for multiple items with a single item being reactivated for each of the four to eight gamma cycles that are nested within one theta cycle. Even though support from human subjects is mounting (Lisman and Jensen, 2013), the primary evidence for this hypothesis derives from rat studies (Jensen and Lisman, 2005). Interestingly, in rats these exact same hippocampal frequency bands have also been proposed to form a mechanism through which rats maintain a spatial configuration in WM in order to support spatial navigation (e.g., Buzsáki, 2005; Tort et al., 2009). Though premature, this link is suggestive of a perspective on serial order such as we have outlined in this paper, linking serial order to spatial coding. As such, our mental whiteboard hypothesis may be a specific example of what Buzsáki and Moser (2013) recently theorized, namely that “mechanisms of memory and planning have evolved from mechanisms of navigation in the physical world” (p. 130).

Hence, whereas direct investigation of the neural substrate underlying the mental whiteboard hypothesis of serial order is yet to emerge, there are already some venues to be derived from existing neuroscientific literature that may guide future investigation in this domain.

### Serial order working memory in (clinical) neuropsychology

Above we provided direct and/or indirect empirical support for the here hypothesized link between serial order WM and spatial processing at the behavioral and neural levels. At the level of (clinical) neuropsychology, this link has not been extensively explored as yet. Still, we here outline a number of studies on dyslexia and hemi-neglect that together will demonstrate the viability of our hypothesis in parsimoniously accounting for a rich set of findings in this domain, too.

In developmental dyslexia, deficits have been observed across separate studies in both serial order WM and in spatial attention. Specifically, Martinez Perez et al. (2012) showed verbal serial order WM impairments in dyslectic children, which could not be fully reduced to impaired phonological processing. Further supporting this idea, Hachmann et al. (2014) demonstrated that WM for serial order (and not for item information) for both verbal and non-verbal information is impaired in dyslexia, whereas Franceschini et al. (2012) showed that tests of visual spatial attention in preschoolers predicts future reading acquisition. The latter study supports a causal role of visual spatial attention in dyslexia, though further research is needed to rule out the alternative explanation that these observations can be attributed to efficient use of *external* spatial attention in the scanning of the document to-be-read–thus lacking a direct link to WM (or *internal* attention).

The notion that the link between serial order WM and spatial attention is involved in reading and spelling also receives (tentative) support from neglect dyslexia. Neglect dyslexia is a disorder commonly observed in hemi-spatial neglect patients who suffer from damage to the left or right parietal lobe. Their difficulties are characterized by reading and spelling errors on the contra-lesional side of words, suggesting that words—representations that involve *serially coded* graphemes—are spatially represented (Caramazza and Hillis, 1990). Although this disorder is typically associated with deficits in visuospatial attention *per se*, patients have actually been described who do not show neglect in tasks other than reading (e.g., Costello and Warrington, 1987; Katz and Sevush, 1989; Cubelli et al., 1991). So far, a comprehensive explanation for this dissociation is lacking, but detailed investigation of one such a patient suggests that neglect dyslexia can also be attributed to deficient coding of (abstract) ordinal position (with graded activation over the different positions), when tasks involve orthographic representations (Petrich et al., 2007). A potential explanation that integrates the currently available observations could be that for accurate writing and spelling, both WM and spatial attention are involved: all graphemes are serially represented in a spatial format in WM, and spatial attention is involved when retrieving this information during the writing or spelling process.

Along the same line of reasoning, additional indications for the link between serial order coding in WM and space can be found within the neglect literature. When neglect patients are asked to indicate the midpoint of a numerical interval they keep in mind, they tend to over-estimate the midpoint (e.g., when asked to indicate the midpoint of the interval 1–9 they may respond 7 instead of 5) as if they ignore the small numbers of the interval (e.g., Zorzi et al., 2002). This bias is typically considered as evidence for the long-term representation of numbers taking the form of a mental number line (MNL). However, more recent observations exist that more closely fit with our hypothesis linking serial order WM to spatial processing. Similar to neglect dyslexia, consistent double dissociations between number interval neglect and neglect within the perceptual space have been observed (e.g., Doricchi et al., 2005) suggesting the involvement of additional cognitive processes which may be the involvement of (serial order) WM. Indeed, Doricchi et al. (2009) showed that rightward deviations in number interval bisection in (right-brain-damaged) neglect patients are correlated both with spatial WM (i.e., Corsi block span) and verbal WM deficits (i.e., digit span). This may be explained by the fact that all these tasks share a common serial order component. Second, van Dijck et al. (2011) reported indications from a left-brain-damaged neglect patient for the notion that (serial) verbal WM supports numerical tasks that are typically linked to a spatial representation of numerical magnitudes. Again, these findings can be easily explained within the here-proposed mental whiteboard hypothesis. After all, for accurate number interval bisection, an ordered series on information (numbers) needs to be maintained, upon which controlled attentional processes operate to obtain a correct response (see Fias et al., 2011, for a detailed elaboration on this idea).

As with the behavioral support outlined above, we realize that each separate finding here can be easily accounted for in various ways; however, we believe that “the bigger picture” that is derived from these findings can be parsimoniously accounted for by the notion that serial order coding occurs within a spatially defined medium.

## Directing future research

In order to contribute to future empirical efforts in this domain, we here close the paper with a number of challenges that we feel are important to further substantiate the mental whiteboard hypothesis.

### Behavioral level

At the *behavioral level*, various empirical questions deserve to guide future research efforts in order to further specify the mental whiteboard hypothesis. First, we need to explore whether the internal spatial code that is derived at the moment of WM retrieval (and which we measure in our paradigms by its interaction with external spatial cues) derives from the direction of the last attention shift between two locations in internal space—or from the location within the overall internal space where attention is focused on that moment. Hence, will shifting attention from the last to the second last item in a long WM sequence provide a right spatial code (as attention is moving through the right-side of space) or a left spatial code (as the specific shift is leftwards from the last to second last item)?

Second, it will need to be explored to what extent the interaction between serial order and spatial attention is modality-independent: Can it also be observed in the visuospatial domain? This remains to be tested, for example, in a design similar to what we employed in the studies by van Dijck et al. (2013, 2014). There are already some indications for the notion that serial order WM *per se* is domain-general. At the neural level, for example, we already noted above that substantial overlap exists in brain areas that underlie verbal and visuospatial WM tasks (Becker et al., 1999; Nystrom et al., 2000; Ravizza et al., 2004; Majerus et al., 2010, 2014). Additionally, at the behavioral level we want to refer to a study by Depoorter and Vandierendonck (2009). They employed a dual-task methodology in which a secondary short-term memory task (verbal vs. visuospatial items; order vs. item memory task) was performed in the retention interval (i.e., the time between presentation and recall) of another, primary short-term memory task. The most important finding for current purposes was that recall performance on the primary task was impaired when both the primary and the secondary tasks involved an order component, irrespective of the modality of the stimulus materials. This is behavioral support for a domain-general serial order WM, in line with earlier suggestions along this line based on similarities between observations for verbal and spatial serial memory (Jones et al., 1995; Smyth, 1996; for a review see Hurlstone et al., 2014).

Third, in our studies on the link between serial order and spatial processing (van Dijck and Fias, 2011; van Dijck et al., 2013, 2014) we capitalized on the left-right dimension. However, as already described above, the mental whiteboard hypothesis postulates flexibility in the precise spatial configuration that is employed—with the only restriction being that it entails a well-arranged, orderly continuum. Along this line, the study by Dutta and Nairne (1993) already tells us that the top-bottom dimension can also be involved. As the precise spatial coding and subsequent trajectory of search through WM content may strongly depend on task context, future research is needed to clarify exactly how task context and representation interact.

Fourth, as noted above, WM maintenance is believed to ultimately lead to consolidation in LTM. As such, interference at the level of WM would be predicted to affect the formation of long-term serial representations. One major paradigm to explore this type of representations is the serial reaction time (SRT) task (Nissen and Bullemer, 1987; Abrahamse et al., 2010). In this task, participants respond one by one to the locations of a series of stimuli by pressing spatially corresponding keys on the key-board. Without them being aware of this, the series of stimuli actually consists of a fixed sequence of stimuli (e.g., with a length of ten stimuli) that is repeated over and over again. Despite lacking awareness of this sequential manipulation, decreases in response times and/or error percentages over time indicate (implicit) sequence learning. Shin and Ivry (2002) have explored if and to what extent spatial (a fixed sequence across four different locations) and temporal (a fixed sequence across four response-to-stimulus intervals) sequences can be learned simultaneously. They observed that spatial and temporal sequences were only learned simultaneously when they were perfectly correlated (i.e., both had equal sequence length of items). When uncorrelated, only the spatial sequence was learned. This type of finding could tentatively be explained by assuming shared systems for both the coding of spatial locations and the coding of ordinal response-to-stimulus interval (i.e., ranked from short to long).

Fifth, at what stage(s) is spatial processing involved in serial order WM? If we take the analog of the whiteboard then it would be more or less implicitly assumed that encoding, maintenance and retrieval (searching across the whiteboard) are all driven by spatial processing. Indeed, this also makes most sense from a logical perspective: why would spatial attention be involved in retrieving information that was not initially coded and maintained in a spatial format? However, this is not yet exclusively supported by available studies (Kirsner and Brown, 1981; van Dijck et al., 2013, 2014). Recent evidence is suggestive for spatial processing during retrieval. Ginsburg et al. (2014) asked participants to memorize a sequence of five numbers in correct serial order and presented during the retention interval numbers that were part of the sequence as well as other numbers for classification (parity judgment or magnitude comparison). Only when the participants were instructed to limit the classification task to numbers from the WM sequence (i.e., verifying its presence in WM) an interaction between the side of response (left or right) and position in the sequence was observed. When the instructions were to classify all numbers presented during the retention interval (no explicit WM retrieval is needed) no such interaction was observed. The roles of encoding and maintenance remain even more elusive up to now. Indirect evidence for the latter has recently been provided by Fischer-Baum and Benjamin (2014) who showed that the recall of serial order information was more accurate when during the encoding phase, the WM items progressed from left to right compared to situation where they progressed in a right to left fashion. Still, future research is required to sort out the involvement of spatial processing across the various stages.

Sixth, what is the role of chunking in the studies by van Dijck et al. (2013, 2014) discussed above? This is an important issue as these studies are the primary behavioral support for the hypothesis that serial order WM is grounded in spatial attention. In all these studies brief sequences were used of only two to four single items each. As this falls within the range of WM limitations for most people, there is no way to determine if these items were chunked together (in sets of either two or four items) or whether each item was represented separately. It is thus an open question if spatial attention is involved merely when searching within a single chunk, or whether the same processes apply to searching across separate items and/or chunks. This needs to be explored in future studies.

### Neuro-imaging and neuropsychological level

At the level of *neuro-imaging* it will be crucial to further outline the role of the IPS in serial order coding. As mentioned above, earlier work has indicated a link between verbal serial order and selective attention, but it did not allow for making any claims about the precise nature of selective attention involvement; hence, whether it concerns *spatial* selective attention formerly still remains to be explored. Possibly, future research can combine the standard Posner cuing paradigm with the adapted version employed by van Dijck et al. (2013), and explore overlap in brain areas.

Our review above points to two areas—the hippocampus and the IPS—whose involvement in serial order and/or spatial processing is fairly well established. From the mental whiteboard hypothesis, the next questions are how and/or when exactly each of these areas is involved, and how they are functionally linked together. It could be speculated that (spatially defined) serial order coding *per se* is tightly linked to hippocampal mechanisms, whereas the IPS contributes controlled and visuospatial imagery processes that underlie the attentional search across serial order representations. This hypothesis could be a starting point for future empirical work in this domain.

Finally, at the level of *clinical neuropsychology*, definite support for the hypothesis that serial order WM is grounded in spatial attention systems is currently lacking. At the moment, contributions of spatial attention to (verbal) serial order (impairments), and vice versa, are hardly explored in (clinical) neuropsychology, and we here call for a more rigorous exploration of this interesting link. Not so much with an eye on supporting our hypothesis, but primarily for the purpose of understanding neurological problems and future opportunities for rehabilitation in especially neglect patients, dyslexia and individuals with impaired verbal WM. For example, it has recently become clear that in dyslexia specific selective deficits in serial order processing exist (Trecy et al., 2013), and our hypothesis may provide a promising avenue for rehabilitation in pointing at spatial attention as an underlying mechanism. It is a missed chance that attentional processing, and particularly spatial attention, is rarely explored in patients with verbal STM deficits—just like pure serial order is hardly tested in spatial neglect.

We would like to point out that our mental whiteboard hypothesis also has methodological implications for (clinical) neuropsychology. The Corsi block tapping task, which today is still one of the most used tasks in assessing visuospatial WM capacity in specific patient populations, involves the simultaneous maintenance of both location and serial order information. As such, it may tax two highly connected systems of external and internal spatial attention. In close resemblance of the studies mentioned in the previous section, then, spatial attention for location information may even interfere with spatial attention for serial order information. This interference is not desirable for a task that aims to provide a clean measure of visuospatial WM capacity. Moreover, the amount of interference may depend on the extent to which serial order and location information align with each other—either on the left-to-right or the top-to-bottom dimension–in a particular spatial block configuration, and the latter varies across studies. We believe that for this domain it is crucial to disentangle item and serial order information depending on the issue that one aims to tackle. Similar concerns may be relevant for other visuospatial WM tasks as well.

## Conclusion

Over the last decades, the attention-based accounts of WM have become increasingly popular. The mental whiteboard hypothesis provides a promising avenue for incorporating serial order within these accounts by grounding it in the spatial attention system. As such, it adds to the overall plausibility of an attention-based WM account. At the same time, this hypothesis provides a candidate mechanism to substantiate the cognitive nature of position markers, a major but currently underspecified concept in serial order models. Still, the mental whiteboard hypothesis currently remains underspecified, and future research is needed to turn this hypothesis into a solid theoretical account.

## Conflict of interest statement

The authors declare that the research was conducted in the absence of any commercial or financial relationships that could be construed as a potential conflict of interest.
